# Association between Subclinical Hypothyroidism and Adverse Pregnancy Outcomes in Assisted Reproduction Technology Singleton Pregnancies: A Retrospective Study

**DOI:** 10.3390/jcm13175137

**Published:** 2024-08-29

**Authors:** Yuichiro Narita, Hiroyuki Tsuda, Eri Tsugeno, Yumi Nakamura, Miho Suzuki, Yumiko Ito, Atsuko Tezuka, Tomoko Ando

**Affiliations:** Department of Obstetrics and Gynecology, Japanese Red Cross Aichi Medical Center Nagoya Daiichi Hospital, Nagoya 453-8511, Japan; yuichiro0202@gmail.com (Y.N.); eri.uchiyama.56@gmail.com (E.T.); yuim0201@gmail.com (Y.N.); miho418miho@gmail.com (M.S.); yum.ito.jrc@gmail.com (Y.I.); a_atsuko_sadaka@yahoo.co.jp (A.T.); ando-tm@nagoya-1st.jrc.or.jp (T.A.)

**Keywords:** ART, preterm birth, subclinical hypothyroidism, thyroid stimulating hormone, levothyroxine therapy

## Abstract

**Background/Objectives**: Women with subclinical hypothyroidism (SCH) were reported to be at an increased perinatal risk. We aimed to investigate the relationship between SCH and perinatal outcomes in singleton pregnancies resulting from assisted reproduction technology (ART). **Methods**: We retrospectively examined the perinatal outcomes of ART singleton pregnancies in women who underwent thyroid function screening before conception and delivered at our hospital from January 2020 to July 2023. We defined SCH as thyroid-stimulating hormone (TSH) levels > 2.5 mU/L and normal free T_4_ levels. The patients were categorized into three groups: normal thyroid function (group A), SCH without levothyroxine therapy (group B), and SCH with levothyroxine therapy (group C). The risks of preterm birth, preeclampsia, fetal growth restriction, manual placental removal, and blood loss at delivery were compared among the three groups. **Results**: Out of the 650 ART singleton deliveries, 581 were assigned to group A, 34 to group B, and 35 to group C. The preterm birth rate at <34 weeks was significantly higher in group B and significantly lower in group C than in group A. The rate of preterm delivery at <34 weeks increased in correlation with TSH levels. Levothyroxine therapy was the significant preventive factor for preterm birth at <34 weeks. **Conclusions**: The preterm birth rate before 34 weeks was significantly higher in the SCH group. Levothyroxine therapy is a significant protective factor against preterm birth before 34 weeks. Universal screening for thyroid function and appropriate hormone therapy in pregnant women may help reduce perinatal risks, including preterm birth.

## 1. Introduction

Thyroid function studies using the Japanese adult general health examination system documented thyroid dysfunction in approximately 10% of cases, with subclinical hypothyroidism (SCH) accounting for half of the cases [1]. This finding suggests that many pregnant women may have undiagnosed SCH. SCH is defined as normal free T_4_ levels and elevated serum thyroid-stimulating hormone (TSH) levels. It is observed in 2.0–2.5% of screened pregnant women, according to reports from iodine-sufficient areas of the United States [2,3].

Some [4,5,6,7], but not all [8,9], studies have demonstrated that women with SCH have a higher perinatal risk of severe preeclampsia, preterm delivery, placental abruption, neonatal respiratory distress syndrome, and/or pregnancy loss than do euthyroid women. One meta-analysis from 19 cohort studies reported an odds ratio (OR) of 1.29 (95% confidence interval [CI], 1.01–1.64) for preterm birth [10], and another meta-analysis reported an OR of 1.53 (95% CI, 1.09–2.15) for preeclampsia [6]. Interestingly, preterm delivery rates increase with TSH levels as follows: 5.4% of pregnancies with TSH levels between 4 and 6 mU/L, 7.8% between 6 and 10 mU/L, and 11.4% with >10 mU/L [11]. Furthermore, limited data suggest that perinatal outcomes are worse in women undergoing in vitro fertilization (IVF) if their preconception TSH levels are >2.5 mU/L. In one study of pregnancies after IVF, 150 deliveries with a preconception TSH level < 2.5 mU/L resulted in higher gestational age and birth weight than 45 deliveries with a TSH level > 2.5 mU/L [12].

Because overt or SCH is thought to be associated with pregnancy complications and thyroid testing is common and easy to perform, attention has been focused on the utility of screening for thyroid dysfunction in all pregnant women. To our knowledge, no high-quality studies have used a universal screening for thyroid function in pregnant women. The advantages and disadvantages of screening thyroid function in all pregnant women in early pregnancy remain inconclusive. This is because there are insufficient data to show not only the effects of thyroid disease on pregnancy but also the benefits of hormone therapy [13,14]. In Japan, universal screening for thyroid dysfunction in asymptomatic pregnant women is rare; however, universal screening for thyroid dysfunction is common in women initiating fertility treatment. This study aimed to examine the association between the presence of SCH and perinatal outcomes in assisted reproduction technology (ART) pregnancies using preconception thyroid function screening and the effects of thyroid hormone replacement (levothyroxine sodium hydrate) during pregnancy.

## 2. Materials and Methods

### 2.1. Participants

**Figure 1:**
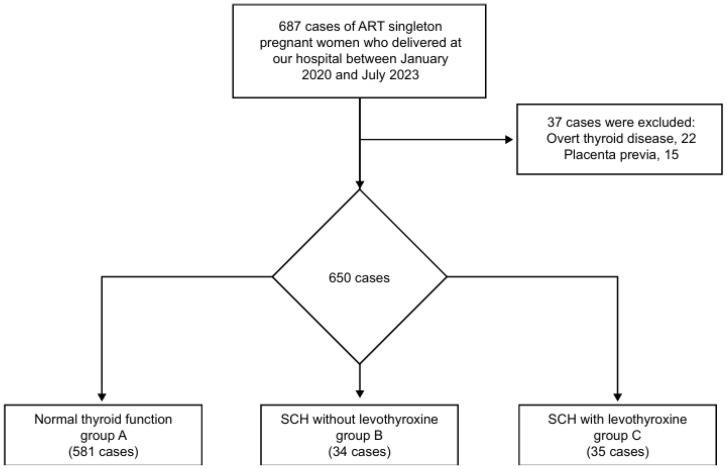
Study flowchart of patient enrollment.

In this retrospective cohort study, we analyzed the perinatal outcomes of pregnancies delivered at the Japanese Red Cross Nagoya Daiichi Hospital, Nagoya, Japan, between January 2020 and July 2023. This study included ART singleton pregnancies in which thyroid function screening was performed before conception (n = 687). Among them, we excluded 22 cases of overt thyroid disease (n = 22) and 15 cases of placenta previa (n = 15) (Figure 1). 

This study was approved by the Ethics Committee of our hospital (approval number: 2023-070).

### 2.2. Data Collection and Definition

In this study, we defined SCH as preconception TSH levels > 2.5 mU/L and normal free T_4_ levels. Patients with preconception TSH levels < 2.5 mU/L were assigned to group A (normal group). Patients with SCH were categorized into two groups: women who took levothyroxine sodium hydrate before and throughout pregnancy (group C) and those who did not (group B). In group C, after diagnosing SCH, patients were treated by an endocrinologist with levothyroxine sodium hydrate (approximately 0.5 µg/kg/day) and followed up regularly (every 2–4 weeks) for TSH and free T4 levels. Doses were increased (12–25 µg/day) until the TSH level fell below 2.5 mU/L. Once the target level was achieved, the maintenance dose was continued until the end of pregnancy (TSH and free T4 levels were monitored every 4–6 weeks). Moreover, all patients in group C had normalized TSH levels at the time of pregnancy. The following maternal and perinatal data were obtained from medical records: age, parity, body mass index before pregnancy, history of abortion, history of previous cesarean deliveries, gestational weeks at delivery, preterm birth (at <37 weeks, <34 weeks, <32 weeks, and <28 weeks), preeclampsia, fetal growth restriction (FGR), blood loss at delivery, transfusion, manual placental removal, and the value of TSH before pregnancy. We also obtained the birth weight of the newborns. Preeclampsia was defined as the appearance of gestational hypertension, proteinuria, and/or signs of end-organ impairment during pregnancy [15]. FGR was defined as neonatal body weight at birth <−1.5 than the standard deviation for gestational age in Japan [16].

### 2.3. Statistical Analyses

All statistical analyses were performed using EZR (v. 1.37, Saitama, Japan). The Shapiro–Wilk test was used to analyze the normality of the data. Continuous variables among the three groups were compared using the Kruskal–Wallis test. The Mann–Whitney U test was used for nonparametric comparisons during the post hoc analysis. Nominal data were analyzed using Fisher’s exact test. In multivariate analysis, maternal age, body mass index, parity, preeclampsia, FGR, history of abortion, levothyroxine therapy, and TSH levels were selected as variables associated with preterm birth. *p* < 0.05 was considered statistically significant.

## 3. Results

Among the 650 ART singleton deliveries in this study, 581 were assigned to group A, 34 to group B, and 35 to group C (Figure 1). The three groups did not significantly differ in terms of age, body mass index, history of previous cesarean deliveries, blood loss at delivery, manual placental removal, transfusion, preeclampsia, FGR, history of abortion, and neonatal body weight (Table 1).

Expectedly, the TSH levels were significantly lower in group A than in groups B and C (*p* < 0.001). After the initiation of levothyroxine therapy in group C, the TSH levels were 1.11 (±0.60) mU/L. The rate of preterm birth at <34 weeks was significantly higher in group B (14.7%) and significantly lower in group C (0%) than in group A (*p* = 0.046). Group C had no cases of preterm delivery under 34 weeks. The preterm birth rate at <37 weeks was lower in group C (5.7%) than in groups A and B; however, the difference was not significant (*p* = 0.059) (Table 1).

When 69 patients diagnosed with SCH (groups B and C) were examined for the rate of preterm delivery at <34 weeks per TSH level, the rates were 1/30 (3.3%) at TSH levels of 2.5–3 mU/L, 2/22 (9.1%) at 3–4 mU/L, and 2/17 (11.8%) at >4 mU/L. The rate of preterm delivery at <34 weeks increased according to the TSH levels, but the difference was not statistically significant.

The results of multivariate analysis of the risk of preterm birth at <37 weeks are shown in Table 2.

When performing the multivariate analysis, we included maternal age, body mass index, parity, preeclampsia, FGR, history of abortion, levothyroxine therapy, and TSH levels as variables to calculate the OR. Preeclampsia (OR = 4.98) and FGR (OR = 3.31) were significant risk factors for preterm birth at <37 weeks; however, levothyroxine therapy and TSH levels did not affect the risk of preterm birth at <37 weeks.

The results of the multivariate analysis of the risk of preterm birth at <34 weeks are shown in Table 3.

In the multivariate analysis, we included maternal age, body mass index, parity, preeclampsia, FGR, history of abortion, levothyroxine therapy, and TSH level as variables to calculate the OR. The significant risk factor for preterm birth at <34 weeks was preeclampsia (OR = 3.65; 95% CI, 1.65–8.09), and the significant preventive factor was levothyroxine therapy (OR = 0.117; 95% CI, 0.015–0.948).

Of the 69 patients with TSH levels > 2.5 mU/L in this study, thyroid peroxidase (TPO) antibodies were measured in 44 patients (63.8%). TPO antibodies were positive in 4 out of 19 patients (21.1%) in group B and 6 out of 25 patients (24%) in group C, with no significant difference between the two groups (*p* = 0.817).

The results of multivariate analysis assessing the impact of TSH on perinatal outcomes are shown in Table S1. TSH levels were not identified as an independent risk factor for various perinatal complications, including preterm birth, preeclampsia, FGR, cesarean delivery, and the need for transfusion.

## 4. Discussion

We investigated the effects of SCH and its preconception treatment on the perinatal outcomes of ART singleton pregnancies, all of which were screened for thyroid function prior to conception. The preterm birth rate at <34 weeks was significantly higher in patients with SCH; however, it was significantly lower in patients with SCH treated with levothyroxine therapy before pregnancy. The rate of preterm delivery at <34 weeks increased according to TSH levels. The multivariate analysis revealed that levothyroxine therapy was a significant protective factor against preterm birth at <34 weeks.

Women with SCH have a higher risk of preeclampsia and preterm delivery than euthyroid women [5,6,10]. However, in the present study, we found that the rate of preterm birth at <34 weeks was significantly higher in patients with SCH and was not significantly different for preterm birth (<37, <32, and <28 weeks) and preeclampsia. The results may be because our study was limited to cases of singleton ART pregnancies. In general, women undergoing infertility treatment appear to have a small but statistically significant increase in risk for preterm birth, low birth weight, and severe maternal morbidity (such as preeclampsia, antepartum hemorrhage, need for transfusion, thrombotic embolism, and disseminated intravascular coagulation) [17,18,19]. Therefore, the background factors of ART pregnancy may have influenced these results. Limited data also suggest that in women undergoing IVF, cases with preconception TSH levels > 2.5 mU/L may result in a lower gestational age at delivery and a lower birth weight [12]. However, other pregnancy outcomes, such as the rate of preterm birth, the rate of FGR, preeclampsia, blood loss during delivery, and the need for transfusion, may not have been examined. To our knowledge, there are no other studies on the association between SCH and adverse outcomes in ART pregnancies, and we believe that the present study provides new insights. In addition, the rate of preterm delivery at <34 weeks increased according to the TSH level but was not significant in the present study. The results are significantly interesting, but the limited number of cases did not allow for significant differences. Previous studies have reported that TSH levels correlate with preterm birth rates [11], but there are no high-quality data stratified by cutoff of the TSH level, presence of antithyroid antibodies, or treatment for SCH. Thus, future studies are required.

Screening for hypothyroidism in asymptomatic pregnant women during early pregnancy remains controversial. In prospective trials, even with universal screening for thyroid function, there was no improvement in pregnancy outcomes compared with a targeted or no screening group [20]. In a randomized trial, >4500 women in their first trimester of pregnancy participated. They were randomly assigned to either a universal screening group or a case-finding group [21]. Overall, the total number of adverse outcomes was similar between the case-finding and universal screening groups. However, secondary analysis revealed that low-risk women diagnosed with SCH and treated with thyroid hormone therapy in the universal screening group had 57% fewer adverse outcomes (preterm birth, preeclampsia, gestational diabetes, and miscarriage) than low-risk women diagnosed with SCH but not treated in the case-finding group [21]. In addition, it has been suggested that universal screening may show higher cost-effectiveness [22,23]. As mentioned above, universal screening of thyroid function in pregnant women has its advantages and disadvantages, but the results of our study suggest that, although limited to ART pregnancies, preconception screening and treatment for SCH may contribute to improved perinatal outcomes. In the future, universal screening for thyroid function and proper hormone therapy in pregnant women may contribute to reducing perinatal risks, including preterm birth.

There are no established criteria for the indications for SCH treatment in pregnant women. In a multicenter trial, 677 pregnant women with SCH (median TSH, 4.4 mU/L; free T4, normal) were randomized to levothyroxine therapy or placebo group [24]. Levothyroxine treatment had no significant effect on maternal or fetal outcomes, such as preterm delivery, preeclampsia, gestational hypertension, and miscarriage, and there was no interaction effect with TPO antibody positivity. In a meta-analysis of nine randomized controlled trials and 13 cohort studies, there was no benefit of SCH treatment on pregnancy outcomes [9]. However, evaluation of antithyroid antibodies is also important in women diagnosed with SCH [4]. In a systematic review by the American Thyroid Association (ATA) regarding pregnancy-specific complications, although there is clearly a higher risk in TPO-positive women with TSH > 2.5 mU/L, the risk was not constant in TPO-negative women, even at significantly higher TSH levels (>5–10 mU/L) [25]. In a trial of 131 TPO antibody-positive women diagnosed with SCH, levothyroxine replacement significantly reduced the rate of preterm delivery, especially in women with TSH ≥ 4 mU/L [26]. At present, it is uncertain whether thyroid hormone replacement therapy reduces perinatal risk in women with SCH. A recent meta-analysis suggested that high levels of TPO antibodies, even in euthyroid pregnant women, could adversely influence pregnancy outcomes after ART [27]. In contrast, no significant differences were observed in pregnancy outcomes following fresh or frozen embryo transfer in euthyroid patients with TPO and/or antithyroglobulin antibodies [28]. Therefore, the perinatal risk of antithyroid antibodies alone in euthyroid patients remains inconclusive. In the present study, we demonstrated that levothyroxine therapy could reduce the risk of preterm birth at <34 weeks among SCH patients with TSH ≥ 2.5 mU/L. However, complete data on anti-TPO antibodies were not available; therefore, further studies are required in the future.

Furthermore, in the present study, the diagnostic criterion for SCH was defined as TSH level > 2.5 mU/L for the following reasons. First, the study included ART pregnancies. Pregnancy outcomes for women undergoing IVF may be worse among those with preconception TSH levels > 2.5 mU/L [12]. Second, the treatment goal for SCH should be to achieve a TSH level of ≤2.5 mU/L. Finally, according to the ATA guidelines, levothyroxine therapy should be considered for TPO antibody-positive women with TSH levels > 2.5 mU/L. Moreover, we decided to use the cutoff of 2.5 mU/L because complete data on TPO antibodies were not available. Therefore, the interpretation of our findings is that levothyroxine therapy with TSH levels > 2.5 mU/L, regardless of the presence of autoantibodies, can reduce the risk of preterm birth at <34 weeks.

This study included cases of ART singleton pregnancies in which thyroid function screening was performed before fertility treatment. The SCH rate was higher in the infertile women than in the control women (healthy women with confirmed fertility) (13.9% vs. 3.9%) [29]. A recent study demonstrated that pregnant women with singleton ART with a history of abortion or spontaneous abortion were more likely to have thyroid-related diseases [30]. In Japan, universal screening of thyroid function is commonly performed before fertility treatment. In addition, all patients in group C were already receiving appropriate levothyroxine therapy by an endocrinologist at the time of conception and were appropriately managed for SCH throughout their pregnancy. The strength of this study is that the patients’ backgrounds were well-established. Second, because it is a single-center study, there is consistency in pregnancy management and treatment policies, and a certain quality of care is maintained. Our institution follows the Guidelines for Obstetrical Practice in Japan and provides standardized care. The limitations of this study include the following: inclusion of only singleton data from ART pregnancies; the absence of data on placental abruption, neonatal respiratory distress syndrome, and/or pregnancy loss among adverse pregnancy outcomes; the absence of data on the prognosis of the child, such as respiratory disorders and cognitive function; and the absence of data on anti-TPO antibodies. Another limitation is that the criteria for levothyroxine therapy (groups B and C) in patients with SCH are unknown. Finally, the number of cases examined in this study was limited, especially in the SCH groups (34 and 35 cases in groups B and C, respectively); therefore, it is possible that statistical differences could not be detected. This study has the following inherent limitations: limitations inherent to the study design, the potential for selection bias, and the inability to generalize the findings to different populations. Therefore, large-scale studies are required in the future.

In conclusion, the rate of preterm birth at <34 weeks is significantly higher in patients with SCH; however, it is significantly lower in patients with SCH treated with levothyroxine therapy before and during pregnancy. Moreover, levothyroxine therapy is a significant protective factor against preterm birth at <34 weeks. These data provide valuable information for future clinical practice. Universal screening of thyroid function and proper hormone therapy in all pregnant women may reduce perinatal risks, including preterm birth. Further large-scale studies are warranted to estimate the perinatal risk of SCH, including data on antithyroid antibodies and the effects of levothyroxine therapy on perinatal risk, and to set cutoff values for appropriate therapeutic interventions.

## Figures and Tables

**Table 1:** Maternal characteristics and perinatal outcomes of this study.

	Group A (n = 581)	Group B (n = 34)	Group C (n = 35)	*p*-Value
Maternal age (years) *	36.5 (±4.3)	36.6 (±4.7)	36.5 (±3.8)	0.988
Pre-pregnant BMI (kg/m^2^) *	21.5 (±3.4)	22.0 (±2.6)	21.8 (±2.8)	0.634
History of abortion	218/581 (37.5%)	11/34 (32.4%)	8/35 (22.9%)	0.194
Preterm birth at <37 weeks	106/581 (18.2%)	9/34 (26.5%)	2/35 (5.7%)	0.059
Preterm birth at <34 weeks	36/581 (6.2%)	5/34 (14.7%)	0/35 (0%)	0.046
Preterm birth at <32 weeks	32/581 (5.5%)	1/34 (2.9%)	0/35 (0%)	0.411
Preterm birth at <28 weeks	16/581 (2.8%)	0/34 (0%)	0/35 (0%)	1
Preeclampsia	56/581 (9.6%)	3/34 (8.8%)	4/35 (11.4%)	0.945
Fetal growth restriction	23/581 (4.0%)	1/34 (2.9%)	0/35 (0%)	0.768
Cesarean delivery	272/581 (46.8%)	15/34 (44.1%)	19/34 (54.3%)	0.641
Blood loss at delivery (mL) *	812 (±676)	970 (±716)	788 (±406)	0.393
Manual placental removal	53/581 (9.1%)	3/34 (8.8%)	5/35 (14.3%)	0.591
Transfusion	33/581 (5.7%)	1/34 (2.9%)	0/35 (0%)	0.374
Neonatal birth weight (g) *	2901 (±1339)	2819 (±640)	3064 (±475)	0.705
TSH value (mU/L) *	1.36 (±0.57)	4.02 (±2.47)	3.55 (±1.07)	<0.001

BMI, body mass index; TSH, thyroid-stimulating hormone. * Median (range).

**Table 2:** Multivariate logistic regression analysis for preterm birth at <37 weeks.

	Adjusted OR	95% CI	*p*-Value
Maternal age	1.00	0.956–1.05	0.880
Pre-pregnant BMI	1.00	0.939–1.07	0.991
Nulliparous	0.95	0.602–1.50	0.822
History of abortion	0.97	0.621–1.52	0.899
Preeclampsia	4.98	2.81–8.81	<0.001
Fetal growth restriction	3.31	1.31–8.34	0.011
Levothyroxine therapy	1.36	0.378–1.36	0.230
TSH value	1.05	0.485–2.29	0.895

BMI, body mass index; TSH, thyroid-stimulating hormone; OR, odds ratio; CI, confidence interval.

**Table 3:** Multivariate logistic regression analysis for preterm birth at <34 weeks.

	Adjusted OR	95% CI	*p*-Value
Maternal age	1.00	0.926–1.07	0.915
Pre-pregnant BMI	1.03	0.942–1.13	0.506
Nulliparous	1.20	0.567–2.53	0.638
History of abortion	1.20	0.606–2.40	0.595
Preeclampsia	3.65	1.65–8.09	0.001
Fetal growth restriction	2.92	0.877–9.74	0.081
Levothyroxine therapy	0.117	0.015–0.948	0.044
TSH value	2.18	0.750–6.36	0.152

BMI, body mass index; TSH, thyroid-stimulating hormone; OR, odds ratio; CI, confidence interval.

## Data Availability

The data in the manuscript will not be deposited. However, upon request, we will submit data (deidentified participant data) that support the findings of this study.

## Supplementary Materials

The following supporting information can be downloaded at: https://www.mdpi.com/article/10.3390/jcm13175137/s1, Table S1: Multivariate logistic regression analysis assessing the impact of TSH on perinatal outcomes.

## References

[1] Kasagi K., Takahashi N., Inoue G., Honda T., Kawachi Y., Izumi Y. (2009). Thyroid function in Japanese adults as assessed by a general health checkup system in relation with thyroid-related antibodies and other clinical parameters. Thyroid.

[2] Allan W.C., Haddow J.E., Palomaki G.E., Williams J.R., Mitchell M.L., Hermos R.J., Faix J.D., Klein R.Z. (2000). Maternal thyroid deficiency and pregnancy complications: Implications for population screening. J. Med. Screen..

[3] Klein R.Z., Haddow J.E., Faix J.D., Brown R.S., Hermos R.J., Pulkkinen A., Mitchell M.L. (1991). Prevalence of thyroid deficiency in pregnant women. Clin. Endocrinol..

[4] Liu H., Shan Z., Li C., Mao J., Xie X., Wang W., Fan C., Wang H., Zhang H., Han C. (2014). Maternal subclinical hypothyroidism, thyroid autoimmunity, and the risk of miscarriage: A prospective cohort study. Thyroid.

[5] Lee S.Y., Cabral H.J., Aschengrau A., Pearce E.N. (2020). Associations between maternal thyroid function in pregnancy and obstetric and perinatal outcomes. J. Clin. Endocrinol. Metab..

[6] Toloza F.J.K., Derakhshan A., Männistö T., Bliddal S., Popova P.V., Carty D.M., Chen L., Taylor P., Mosso L., Oken E. (2022). Association between maternal thyroid function and risk of gestational hypertension and pre-eclampsia: A systematic review and individual-participant data meta-analysis. Lancet Diabetes Endocrinol..

[7] Breathnach F.M., Donnelly J., Cooley S.M., Geary M., Malone F.D. (2013). Subclinical hypothyroidism as a risk factor for placental abruption: Evidence from a low-risk primigravid population. Aust. N. Z. J. Obstet. Gynaecol..

[8] Cleary-Goldman J., Malone F.D., Lambert-Messerlian G., Sullivan L., Canick J., Porter T.F., Luthy D., Gross S., Bianchi D.W., D’Alton M.E. (2008). Maternal thyroid hypofunction and pregnancy outcome. Obstet. Gynecol..

[9] Jiao X.F., Zhang M., Chen J., Wei Q., Zeng L., Liu D., Zhang C., Li H., Zou K., Zhang L. (2022). The impact of levothyroxine therapy on the pregnancy, neonatal and childhood outcomes of subclinical hypothyroidism during pregnancy: An updated systematic review, meta-analysis and trial sequential analysis. Front. Endocrinol..

[10] Korevaar T.I.M., Derakhshan A., Taylor P.N., Meima M., Chen L., Bliddal S., Carty D.M., Meems M., Vaidya B., Consortium on Thyroid and Pregnancy—Study Group on Preterm Birth—Study Group on Preterm Birth (2019). Association of thyroid function test abnormalities and thyroid autoimmunity with preterm birth: A systematic review and meta-analysis. JAMA.

[11] Knøsgaard L., Andersen S., Hansen A.B., Vestergaard P., Andersen S.L. (2023). Maternal hypothyroidism and adverse outcomes of pregnancy. Clin. Endocrinol..

[12] Baker V.L., Rone H.M., Pasta D.J., Nelson H.P., Gvakharia M., Adamson G.D. (2006). Correlation of thyroid stimulating hormone (TSH) level with pregnancy outcome in women undergoing in vitro fertilization. Am. J. Obstet. Gynecol..

[13] Blatt A.J., Nakamoto J.M., Kaufman H.W. (2012). National status of testing for hypothyroidism during pregnancy and postpartum. J. Clin. Endocrinol. Metab..

[14] Vaidya B., Hubalewska-Dydejczyk A., Laurberg P., Negro R., Vermiglio F., Poppe K. (2012). Treatment and screening of hypothyroidism in pregnancy: Results of a European survey. Eur. J. Endocrinol..

[15] Watanabe K., Matsubara K., Nakamoto O., Ushijima J., Ohkuchi A., Koide K., Makino S., Mimura K., Morikawa M., Naruse K. (2018). Outline of the new definition and classification of “hypertensive disorders of pregnancy (HDP)”; a revised JSSHP statement of 2005. Hypertens. Res. Pregnancy.

[16] Yoshida S., Unno N., Kagawa H., Shinozuka N., Kozuma S., Taketani Y. (2000). Prenatal detection of a high-risk group for intrauterine growth restriction based on sonographic fetal biometry. Int. J. Gynaecol. Obstet..

[17] Jaques A.M., Amor D.J., Baker H.W.G., Healy D.L., Ukoumunne O.C., Breheny S., Garrett C., Halliday J.L. (2010). Adverse obstetric and perinatal outcomes in subfertile women conceiving without assisted reproductive technologies. Fertil. Steril..

[18] Declercq E., Luke B., Belanoff C., Cabral H., Diop H., Gopal D., Hoang L., Kotelchuck M., Stern J.E., Hornstein M.D. (2015). Perinatal outcomes associated with assisted reproductive technology: The Massachusetts Outcomes Study of Assisted Reproductive Technologies (MOSART). Fertil. Steril..

[19] Murugappan G., Li S., Lathi R.B., Baker V.L., Luke B., Eisenberg M.L. (2020). Increased risk of severe maternal morbidity among infertile women: Analysis of US claims data. Am. J. Obstet. Gynecol..

[20] Lazarus J.H., Bestwick J.P., Channon S., Paradice R., Maina A., Rees R., Chiusano E., John R., Guaraldo V., George L.M. (2012). Antenatal thyroid screening and childhood cognitive function. N. Engl. J. Med..

[21] Negro R., Schwartz A., Gismondi R., Tinelli A., Mangieri T., Stagnaro-Green A. (2010). Universal screening versus case finding for detection and treatment of thyroid hormonal dysfunction during pregnancy. J. Clin. Endocrinol. Metab..

[22] Thung S.F., Funai E.F., Grobman W.A. (2009). The cost-effectiveness of universal screening in pregnancy for subclinical hypothyroidism. Am. J. Obstet. Gynecol..

[23] Dosiou C., Barnes J., Schwartz A., Negro R., Crapo L., Stagnaro-Green A. (2012). Cost-effectiveness of universal and risk-based screening for autoimmune thyroid disease in pregnant women. J. Clin. Endocrinol. Metab..

[24] Casey B.M., Thom E.A., Peaceman A.M., Varner M.W., Sorokin Y., Hirtz D.G., Reddy U.M., Wapner R.J., Thorp J.M., Saade G. (2017). Treatment of subclinical hypothyroidism or hypothyroxinemia in pregnancy. N. Engl. J. Med..

[25] Alexander E.K., Pearce E.N., Brent G.A., Brown R.S., Chen H., Dosiou C., Grobman W.A., Laurberg P., Lazarus J.H., Mandel S.J. (2017). 2017 Guidelines of the American Thyroid Association for the diagnosis and management of thyroid disease during pregnancy and the postpartum. Thyroid.

[26] Nazarpour S., Ramezani Tehrani F., Simbar M., Tohidi M., Alavi Majd H., Azizi F. (2017). Effects of levothyroxine treatment on pregnancy outcomes in pregnant women with autoimmune thyroid disease. Eur. J. Endocrinol..

[27] Zhang S., Yang M., Li T., Yang M., Wang W., Chen Y., Ding Y., Liu J., Xu X., Zhang J. (2023). High level of thyroid peroxidase antibodies as a detrimental risk of pregnancy outcomes in euthyroid women undergoing ART: A meta-analysis. Mol. Reprod. Dev..

[28] Yang X., Qiu S., Jiang W., Huang Z., Shi H., Du S., Sun Y., Zheng B. (2023). Impact of thyroid autoimmunity on pregnancy outcomes in euthyroid women following fresh/frozen–thawed embryo transfer. Clin. Endocrinol..

[29] Abalovich M., Mitelberg L., Allami C., Gutierrez S., Alcaraz G., Otero P., Levalle O. (2007). Subclinical hypothyroidism and thyroid autoimmunity in women with infertility. Gynecol. Endocrinol..

[30] Sun H., Su X., Liu Y., Li G., Liu X., Du Q. (2022). Association between abortion history and perinatal and neonatal outcomes of singleton pregnancies after assisted reproductive technology. J. Clin. Med..

