# Supplementary material for: Association between Subclinical Hypothyroidism and Adverse Pregnancy Outcomes in Assisted Reproduction Technology Singleton Pregnancies: A Retrospective Study
Source: J Clin Med. 2024 Aug 29;13(17):5137. doi: 10.3390/jcm13175137 (PMC11395917; doi:10.3390/jcm13175137)
Supplement: Supplementary file 1 [file jcm-13-05137-s001.zip › jcm-3152046-supplementary.pdf]

Supplementary Material

Table S1. Multivariate logistic regression analysis assessing the impact of TSH on perinatal outcomes.

	Adjusted OR	95% CI	P-value
Preterm birth at <37 weeks*	1.05	0.485–2.29	0.895
Preterm birth at <34 weeks*	2.18	0.750–6.36	0.152
Preterm birth at <32 weeks*	0.89	0.593–1.33	0.561
Preterm birth at <28 weeks*	1.21	0.241–6.12	0.814
Preeclampsia**	1.16	0.875–1.54	0.302
Fetal growth restriction***	0.94	0.534–1.67	0.843
Cesarean delivery****	1.01	0.824–1.24	0.930
Manual placental removal****	1.06	0.782–1.43	0.709
Transfusion****	0.75	0.405–1.38	0.355

*: Variables: maternal age, body mass index, nulliparous, history of abortion, preeclampsia, fetal growth restriction, and levothyroxine therapy
**: Variables: maternal age, body mass index, nulliparous, history of abortion, preterm birth at <37 weeks, fetal growth restriction, and levothyroxine therapy
***: Variables: maternal age, body mass index, nulliparous, history of abortion, preterm birth at <37 weeks, preeclampsia, and levothyroxine therapy
****: Variables: maternal age, body mass index, nulliparous, history of abortion, preterm birth at <37 weeks, preeclampsia, fetal growth restriction, and levothyroxine therapy

TSH, thyroid-stimulating hormone; OR, odds ratio; CI, confidence interval
